# A Manual of Diseases of the Nose and Throat, Including the Nose, Naso-Pharynx, Pharynx, and Larynx

**Published:** 1891-06

**Authors:** 


*A Manual of Diseases of the Nose and Throat, including the Nose, Naso-pharynx, Pharynx, and Larynx.* By PROCTER S. HUTCHINSON, M.R.C.S. London: H. K. LEWIS. 1891.

In the preface to this book the writer acknowledges his indebtedness to numerous authors, of whom he enumerates three, for giving him the ideas which he has put into his work. We cannot help feeling that if he had left it to us to read the writings of the many excellent specialists who have given us the results of years of observation and experience gained from their patients, it would have been better than for him to attempt to help us by putting into our hands the work under review.

There does not appear to us to be any room on our shelves for such a book, seeing that we already possess so much literature on the subject from the pens of both specialists and general physicians and surgeons, and we do not believe that it is always useful to boil down several books into one small Manual merely to secure conciseness; and there is little or nothing of an original character in this effort of the author. It is, on the whole, well illustrated, some of the illustrations being old friends; but a few of the drawings might have been omitted.

### Notes on Preparations for the Sick.

